# Hepatocellular carcinoma and liver metastases: clinical data on a new dual-lumen catheter kit for surgical sealant infusion to prevent perihepatic bleeding and dissemination of cancer cells following biopsy and loco-regional treatments

**DOI:** 10.1186/s13027-015-0006-0

**Published:** 2015-04-10

**Authors:** Francesco Izzo, Raffaele Palaia, Vittorio Albino, Alfonso Amore, Raimondo di Giacomo, Mauro Piccirillo, Maddalena Leongito, Aurelio Nasto, Vincenza Granata, Antonella Petrillo, Secondo Lastoria

**Affiliations:** Abdominal Surgical Oncology and Hepatobiliary Unit, Istituto Nazionale Tumori, IRCCS Fondazione “G. Pascale”, Via M.Semmola, 80131 Naples, Italy; Radiology Unit, Istituto Nazionale Tumori, IRCCS Fondazione “G. Pascale”, Via M.Semmola, 80131 Naples, Italy; Nuclear Medicine Unit, Istituto Nazionale Tumori, IRCCS Fondazione “G. Pascale”, Via M.Semmola, 80131 Naples, Italy

**Keywords:** Hepatocarcinoma, Liver metastases, Dual lumen catheter, Sealant, Locoregional treatments

## Abstract

**Background:**

RFA is a safe and effective procedure for treating unresectable primary or secondary liver malignancies, but it is not without complications. The most common reported complications include abdominal hemorrhage, bile leakage, biloma formation, hepatic abscesses, and neoplastic seeding.

The aim of this study is to evaluate the feasibility of percutaneous use of surgical sealant with a new coaxial bilumen catheter, to prevent the perihepatic bleeding and dissemination of cancer cells through the needle-electrode (neoplastic seeding) or along the needle track.

**Methods:**

We designed a novel dual-lumen catheter to facilitate the optimal application of fibrin sealant after diagnostic and therapeutic percutaneous procedures. Percutaneous RFA has been performed using mask ventilation or neuroleptanalgesia. The main aims of this study, after the ablation procedure, in the treatment of unresectable liver cancer were to prevent major adverse events: a) the perihepatic bleeding; b) dissemination of cancer cells through the needle-electrode and or needle track.

**Results:**

A total of 181 patients were evaluated for this study at National Cancer Institute of Naples from January 2012 to January 2014. The association of blood loss (≤1 g/dl; ≥1 g/dl) with age, gender, histological diagnosis were analyzed. No statistical significance was observed between bleeding and age (p = 0.840), gender (p = 0.607) and histological diagnosis (p = 0,571), respectively.

**Conclusions:**

This study demonstrated that fibrin sealant or other surgical sealant injection, after any locoregional procedure such as biopsy or ablation, could make adverse events even more rare.

## Introduction

Radiofrequency ablation has been widely accepted as an effective modality for treating unresectable hepatocellular carcinoma (HCC) and liver metastases [1-4]. It is a thermo**-**ablative technique, based on the conversion of radiofrequency waves into heat, and thus generating areas of coagulative necrosis and tissue desiccation [5].

RFA is not without complications, which range from 6.3 to 9.5% according to current estimates. The most common reported complications associated with percutaneous RFA include abdominal hemorrhage, bile leakage, biloma formation, hepatic abscesses, and neoplastic seeding [6-8].

The aim of this study is to evaluate the feasibility of percutaneous use of surgical sealant with a new coaxial bilumen catheter, to prevent the perihepatic bleeding and dissemination of cancer cells through the needle-electrode (neoplastic seeding) or along the needle track, after locoregional procedure in the treatment of primary or metastatic liver cancer.

## Patients and methods

The study was approved by the local Ethics Committee.

Written informed consent was obtained from the patient for the study as well as for publication of this report and any accompanying images.

All patients have been clinically assessed and investigated with laboratory tests for liver functions, hepatitis B/C serology, alpha-fetoprotein, carcinoembryonic antigen and Ca19**-**9.

Radiologically, the staging has been performed with a chest X-ray, trans-abdominal ultrasound, followed by a 3-phase contrast computed tomography (CT) scan and/or magnetic resonance (RM). In situations where a malignant nature was uncertain, liver biopsy was performed prior to ablation.

All reported RFA procedures were performed percutaneously, with ultrasound guide.

Based on the literature, selection was based on: 1 or 3 lesions ≤ 3,0 cm, 1 lesion ≤ 5 cm in all cases. The maximum dimension of the tumors was not greater than 5 cm determined by CT scan measurement.

There was no evidence of extra-hepatic disease upon diagnosis. Percutaneous RFA has been performed using mask ventilation or neuroleptanalgesia. If the nature of the lesion has not been confirmed radiologically and biochemically, a liver biopsy through a co-access needle system has been performed immediately prior to ablation. Only malignant nodules were included in the present study. The main aims of this study, after the ablation procedure, in the treatment of unresectable liver cancer were to prevent major adverse events: a) perihepatic bleeding; b) dissemination of cancer cells through the needle-electrode and or needle track.

In our series we did not observe any adverse events, we focused the attention on blood loss to assess any statistical differences for all the variables of interest. The cut-off value for blood loss was less than 1 g/dl and greater/equal than 1 g/dl (<1 g/dl; ≥1 g/dl). The association between blood loss and some covariates was assessed with Chi-Square test; we considered p-value less than 0.05 statistically significant. All statistical analyses were performed with SPSS statistical software version 21 (SPSS inc., Chicago IL, USA).

### Ablation technique

To perform a biopsy and/or percutaneous therapeutic procedure, the appropriate introducer is positioned using US, CT or MR imaging guidance. After removing the core of the introducer, the appropriate needle for the procedure is inserted with the tip positioned at the end of the target tissue, complete ablation of the target tissue is achieved when tissue desiccation results in an increase in the tissue impedance. The amount of energy required to reach complete desiccation and coagulation will be dependent upon the volume of the target tissue and the heat-sink effect of local vascularity. When treatment is completed the needle-electrode is removed and the coaxial, dual-lumen catheter for sealant application is inserted. The sealant is injected into the biopsied or ablated area, along the introducer track, while the introducer is carefully removed.

**Figure 1:**
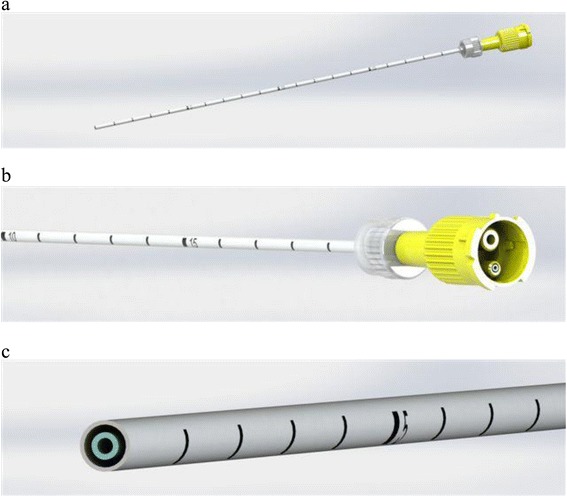
Dual-lumen catheter.** (a)** Design of dual-lumen catheter. **(b)** Detail of the superior Luer-Lock attachment with evidence of the coaxial dual-lumen. **(c)** Internal section of the catheter.

**Figure 2:**
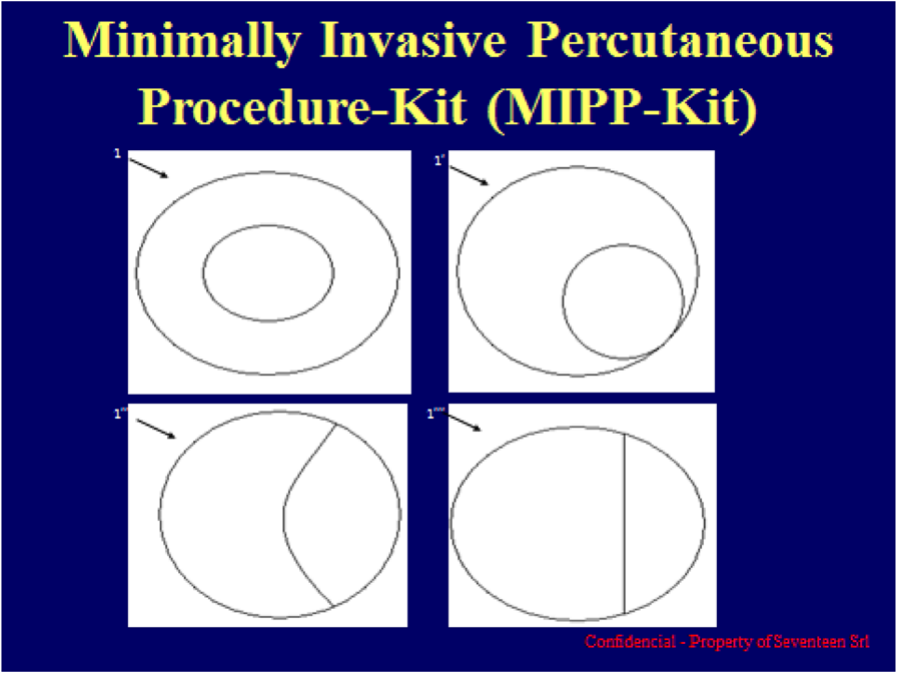
Design of dual-lumen catheter: transverse section showing diameters of lumen.

**Figure 3:**
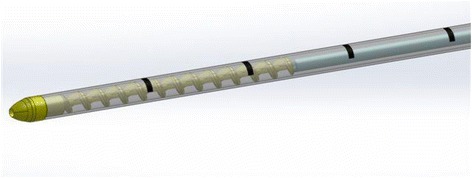
Catheter tip with an inner spiral shape in which two components of the sealant are mixed before the injection.

The kit used in the present study consisted of a 14G introducer, a 15G coaxial dual-lumen catheter, and a needle (Figure 1a,b,c). The introducer (Grimalind® L25) and catheter (20 cm long) were graduated, of the same length (200 mm) and made of radiopaque material (30% barium sulfate). The catheter had a steel core, a sharp and removable tip that was slightly longer than the introducer (210 mm) and a Luer-Lock connection. The needle had an oblique tip to allow easy penetration of the tissue. The catheter composed of Grilfex® ELG 6260 (PEBA) — a radiopaque and semi-rigid material — has two chambers one for each sealant component (Figure 2) and an inner spiral shape in which two components of the sealant are mixed before the injection (Figure 3).

The dual-lumen catheter was designed for the application of Tisseel® (Baxter) and other fibrin sealants or surgical sealants, following percutaneous liver procedures including FNA biopsy and RFA.

**Tisseel**® by Baxter is a 2 component fibrin sealant [9].

- The sealer protein solution contains human fibrinogen and a synthetic fibrinolysis inhibitor, aprotinin, which helps prevent premature degradation of fibrin clot
- The thrombin solution contains human thrombin and calcium chloride

When mixed together, the 2 components combine and mimic the final stages of the body’s natural clotting cascade to form a rubber-like mass that adheres to the wound surface and achieves hemostasis and sealing or gluing of tissues.

Thrombin is a highly specific protease that transforms the fibrinogen contained in Sealer Protein (Human) into fibrin. Fibrinolysis inhibitor, Aprotinin (Synthetic), is a polyvalent protease inhibitor that prevents premature degradation of fibrin. Preclinical studies with different fibrin sealant preparations simulating the fibrinolytic activity generated by extracorporeal circulation in patients during cardiovascular surgery have shown that incorporation of aprotinin in the product formulation increases resistance of the fibrin sealant clot to degradation in a fibrinolytic environment.

Two other surgical sealant has been used in our practice:

- **Bioglue** by CryoLife® [10]
- BioGlue’s two components, purified bovine serum albumin (BSA) and glutaraldehyde, are dispensed by a controlled delivery system. Once dispensed, the two components are fully mixed within the applicator tip. BioGlue, upon application to the tissue at the repair site, creates a flexible mechanical seal independent of the body’s clotting mechanisms.
- BioGlue begins to polymerize within 20 to 30 seconds and reaches full strength within two minutes. CryoLife’s BioGlue syringe is a self-contained, easy to use, disposable delivery system requiring no thawing, reconstitution or manual mixing. The pre-filled syringe is available in 2 mL, 5 mL, and 10 mL volumes to meet the hemostatic needs of the surgeon.

**Beriplast** ®P by Nycomed-Takeda contains [11]:

Fibrinogen (Human), coagulation factor XIII (Human), aprotinin (bovine), thrombin (human), calcium chloride.

The fibrin adhesion system initiates the last phase of the physiological process of blood clotting. By the action of thrombin the fibrinogen is converted to fibrin. The fibrin thus formed is then stabilized by cross-linking by factor XIII thus giving rise to a lattice compact, mechanically stable and with good adhesive characteristics. To avoid an excessively rapid fibrinolysis, the fibrin glue is added with aprotinin.

Fibrinogen and thrombin solutions have different viscosities and therefore different flow rates through the catheter lumen which mean they are not delivered simultaneously and in equal amounts to the injury site leading to inadequate sealant application and clot formation. The aim and innovation was to design a catheter through which the two components would flow at similar rates and would be delivered simultaneously and in equal amounts at the site of injury thus reducing the time and force necessary to inject the sealant.

The first step was to modify the lumen of the catheter based on the density and viscosity of each solution. Using Poiseuille’s law it established that the optimal ratio between the surface of the lumen cross section containing the fibrinogen solution (the more viscous solution) and that of the lumen containing the thrombin solution (less viscous) should be greater than the square root of the ratio of the viscosities of both solutions. The dual-lumen catheter of the present study was then manufactured based on these dimensions. It has now been granted the Italian patent (0001395842) while is pending the international one (PCT/IT2010/000241).

After treatment all patients have been evaluated by the photometric cyanmethemoglobin method at 6 and 12 hours from the procedure and with clinical examination every three months; US and CT were used alternatively every three months from the procedure.

## Results

This is a retrospective study. A total of 181 patients were evaluated for this study at National Cancer Institute of Naples from January 2012 to January 2014. Preoperatively, these patients had a mean age of 70.7 ± 9.2 years (range 42–88), 59% (107) were males and 41% (74) were females. The histological diagnosis was of 133 HCC (73.5%), 43 metastases (23.8%) and 5 cholangiocarcinomas (2.8%). The main demographic and clinical characteristics are shown in Table 1.

**Table 1:** **Descriptive and demographic characteristics**

**Characteristics**	**N (%)**
**Gender**	
Males	107 (59.1)
Females	74 (40.9)
**Histological Diagnosis**	
HCC	133 (73.5)
Metastases	43 (23.8)
Cholangiocarcinomas	5 (2.8)
**Age**	
<= 65 yrs	42 (23.2)
66-75 yrs	80 (44.2)
>75 yrs	59 (32.6)

The association of blood loss (<1 g/dl; ≥1 g/dl) with age, gender, histological diagnosis were analyzed. No statistical significance was observed between bleeding and age (p = 0.840), gender (p = 0.607) and histological diagnosis (p = 0.571), respectively (Table 2).

**Table 2:** **Distribution of selected variables according to Blood Loss**

	**Low blood less <1gr/dl**	**High blood less ≥1 gr/dl**	P value*
**Gender**			0.61
Males	88	19	
Females	63	11	
**Histological Diagnosis**			0.57
HCC	111	22	
Metastases	35	8	
Cholangiocarcinomas	5	0	
**Age**			0.84
<= 65 yrs	36	6	
66-75 yrs	67	13	
>75 yrs	48	11	

*Chi-Square test.

The median length of hospital stay was 1 day.

The clinical examination and US/CT at 1, 3, 6 and 9 months from the percutaneous procedure have not highlighted needle-track seeding in none of the patients.

## Discussion

The two most commonly encountered liver malignancies in our area are hepatocellular carcinoma and colorectal liver metastases. While surgical resection remains the “gold standard” for treating both conditions, only a small proportion of patients are suitable candidates for curative intent hepatectomy [9,12].

This may be due to patient’s comorbidity, poor liver reserve, presence of extrahepatic disease, or it may be related to the number and anatomical location of the tumors. Radiofrequency ablation has become one of the best alternatives in treating these patients who are not candidates for curative liver resection. A systematic review by Sutherland et al. concluded that RFA generally resulted in larger and more complete areas of ablation, and RFA may also be associated with higher survival rates than the other ablative techniques being assessed [1,13,14].

The aim of this study was to prevent the perihepatic bleeding and neoplastic seeding after RFA, using fibrin sealant after the ablation procedure in the treatment of liver cancer, performed at our center.

Fibrin sealant preparations are currently commercially available and are successfully used in clinical practice worldwide.

The percutaneous approach is less invasive and the patients’ length of hospital stay is significantly shorter.

Complications from RF ablation are uncommon. Mulier et al. reviewed 3,670 patients after RFA; mortality and morbidity rates were 0.5% and 8.9%, respectively [8].

Another multicentre survey on 582 hepatic tumors over a 5-year period reported the mortality, major and minor complication rates to be 1.4%, 5.7% and 6.3%, respectively [7].

In 41 papers specifying the total number of patients biopsied and/or treated, the median risk of seeding was 2.29% (range 0–11%) for biopsy group; 1.4% (1.15–1.85%) for PEI when used with biopsy and 0.61% (0–5.56%) for RFA without biopsy, 0.95% (0–12.5%) for RFA with biopsy and 0.72% (0–10%) for liver nodules (including non-HCC nodules) biopsied and ablated [15].

Control of intraoperative hemorrhage is particularly difficult when the site is poorly accessible, in case of altered coagulation parameters and in patients with congenital or acquired bleeding disorders. Parenchymal organs, such as the liver, pose additional problems due to their soft nature and propensity to bleed.

In addition to monitoring vital signs, base deficit, and lactate as well as serial measurements of coagulopathy and hemoglobin (Hgb) concentration are frequently used to evaluate for ongoing hemorrhage. At present, the photometric cyanmethemoglobin method is the most widely used technique for measuring Hgb in the laboratory and is currently the standard criterion, as defined by the International Committee for Standardization in Hematology [16-18].

Fibrin sealants and surgical sealant have been used successfully in a variety of surgical and endoscopic settings to promote hemostasis and tissue sealing [19-22].

The fibrin sealant is indicated to assist hemostasis in adult and pediatric patients (>1 month of age) undergoing surgery when control of bleeding by conventional surgical techniques (such as suture, ligature, and cautery) is ineffective or impractical. This is effective also in heparinized patients and in patients medicated with anti-platelet drugs.

Fibrin sealant is also used to prevent the seeding along the needle track. In fact, needle track seeding in patients treated with percutaneous RFA has raised a great deal of concern. Bonatti et al. reported a case of skin implant metastasis after percutaneous RFA for colorectal liver metastasis [23].

Seeding has also been described after percutaneous RFA for hepatocellular carcinoma performed with a single cooled-tip electrode [24].

There are several hypothesis for this phenomenon: dissemination of tumor cells on retraction of the radiofrequency electrode, tumor cell spread from needle track hemorrhage and cells extruded by an increased intra-tumoral pressure during RFA [25].

**Figure 4:**
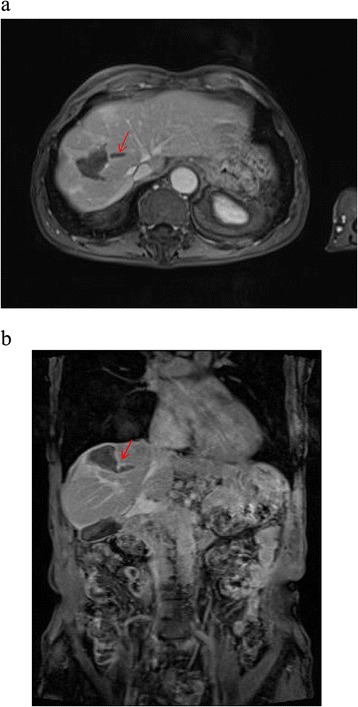
Axial **(a)** and coronal view **(b)** of MRI ViBE sequences after intra venous injection of contrast medium. In addition to the clear post-RFA coagulative necrosis, in the hepatic parenchyma it is visible the needle way, indicated by the arrow.

**Figure 5:**
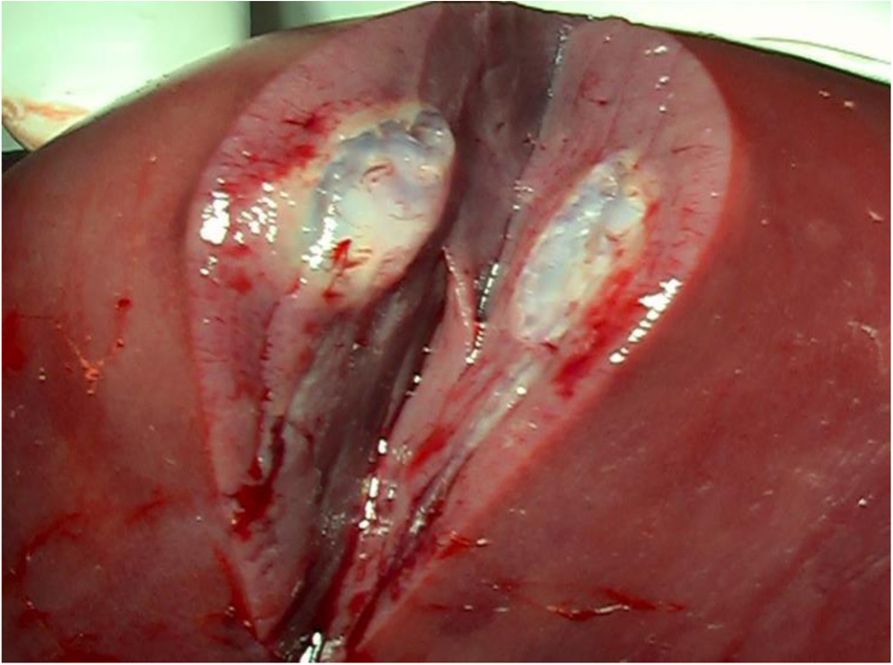
Macroscopic evidence (in a specimens post liver resection) where is evident the presence of the sealant inside the tumor and along the needle-track.

In our study, the procedure was completed in all cases and no laparotomy or angiographic procedure were necessary to achieve hemostasis. The interventions (FNA and RFA) were not associated with complications as anticipated including biloma, hepatic abscess, and bile duct injury. The absence of these complications typically associated with the two interventions, was confirmed by clinical and radiological follow-up (median 18 months) (Figures 4 and 5). Blood tests did not reveal any abnormal coagulation parameters after the procedure. The kit was easy to use and the application of the fibrin sealant did not pose any particular technical problems. The fibrin sealant could be applied correctly: the two sealant components were mixed correctly at 37°C and at the proper time ensuring the optimal application of active hemostatic agent at the treated liver sites.

Optimal administration of the fibrin sealant after biopsy or to the ablated site is frequently difficult to achieve when the sealant is delivered via a catheter as occurs during percutaneous procedures. There is therefore an unmet medical need for a new delivery system that will ensure correct administration of the effective sealant to the surgical site. Our group designed and patented this novel coaxial dual lumen catheter system for the infusion of bi-component hemostatic or sealant agents. The objective was to optimize administration of the fibrin sealant during a range of clinical procedures.

The results of our small-scale retrospective study show that the patented minimally invasive percutaneous procedure-kit (MIPP-Kit) confirm that the kit is significantly easier to use, much less force is require to push the two substances out of the catheter and that it allows more precise delivery of active agents to the site required. Importantly, blockage or clogging of the catheter tip did not occur. Investigators did not report any difficulties in inserting the needle into the site of infusion probably as a result of the fact that the needle was designed with an oblique tip that facilitated easy entry.

## Conclusion

In conclusion, this study demonstrated that RFA is a safe and effective procedure for treating unresectable primary or secondary liver malignancies. The perihepatic bleeding and dissemination of cancer cells through the needle-electrode (neoplastic seeding) depend only on the procedure.

A limit of this study is the lack of a control group; the effectiveness in the prevention of bleeding, as result of the use of fibrin glue, cannot be absolutely proven. Nevertheless comparing our results with our previous clinical experience and the data reported in literature, injecting fibrin sealant or other surgical sealant, after any locoregional procedure like biopsy or ablation, makes these events even more rare.

## Abbreviations

FNA: Fine needle aspiration
RFA: Radiofrequency ablation
HCC: Hepatocellular carcinoma
US: Ultrasound
CT: Computed tomography
MR: Magnetic resonance
MIPP-Kit: Minimally invasive percutaneous procedure-kit

## References

[1] Bruix J, Sherman M (2005). Management of hepatocellular carcinoma. Hepatology.

[2] Wong J, Lee KF, Lee PS, Ho SS, Yu SC, Ng WW (2009). Radiofrequency ablation for 110 malignant liver tumours: preliminary results on percutaneous and surgical approaches. Asian J Surg.

[3] Oshowo A, Gillams A, Harrison E, Lees WR, Taylor I (2003). Comparison of resection and radiofrequency ablation for treatment of solitary colorectal liver metastasis. Br J Surg.

[4] Izzo F, Barnett CC, Curley SA (2001). Radiofrequency ablation of primary and metastatic malignant liver tumors. Adv Surg.

[5] Curley SA (2001). Radiofrequency ablation of malignant liver tumours. Oncologist.

[6] Curley SA, Marra P, Beaty K, Ellis LM, Vauthey JN, Abdalla EK (2004). Early and late complications after radiofrequency ablation of malignant liver tumors in 608 patients. Ann Surg.

[7] De Baère T, Risse O, Kuoch V, Dromain C, Sengel C, Smayra T (2003). Adverse events during radiofrequency treatment of 582 hepatic tumors. AJR Am J Roentgenol.

[8] Mulier S, Mulier P, Ni Y, Miao Y, Dupas B, Marchal G (2002). Complications of radiofrequency coagulation of liver tumours. Br J Surg.

[9] Baxter Monograph on: TISSEEL, Doc. 160973, March 21, 2013. webcite at: http://www.baxter.ca/en/downloads/product_information/TISSEEL_VHSD_PM_EN_21Mar.

[10] CryoLife Manual Instruction on: BioGlue http://www.cryolife.com/images/stories/assets/docs/BG_Surgical_Adhesive_Syringe_IFU_dom.pdf.

[11] Aventis- Behring Product info on: Beriplast –P/ Combi Set http://www.cslsurgery.com/international/beriplast/product/pdf/Wounded_Healing_8_9.pdf.

[12] Lai EC, Fan ST, Lo CM, Chu KM, Liu CL, Wong J (1995). Hepatic resection for hepatocellular carcinoma: an audit of 343 patients. Ann Surg.

[13] Sutherland LM, Williams JA, Padbury RT, Gotley DC, Stokes B, Maddern GJ (2006). Radio-frequency ablation of liver tumours. A systematic review. Arch Surg.

[14] Lencioni RA, Allgaier HP, Cioni D, Olschewski M, Deibert P, Crocetti L (2003). Small hepatocellular carcinoma in cirrhosis: randomized comparison of radio- frequency thermal ablation versus percutaneous ethanol injection. Radiology.

[15] Stigliano R, Marelli L, Yu D, Davies N, Patch D, Burroughs AK (2007). Seeding following percutaneous diagnostic and therapeutic approaches for hepatocellular carcinoma. What is the risk and the outcome? Seeding risk for percutaneous approach of HCC. Cancer Treat Rev.

[16] Tsuei BJ, Hanseman DJ, Blakeman MJ, Blakeman TC, Yang SH, Branson RD (2014). Accuracy of noninvasive hemoglobin monitoring in patients at risk for hemorrhage. J Trauma Acute care Surg.

[17] Van Kampen E, Zijlstra WG (1961). Standardization of hemoglobinometry. II. The hemiglobincyanide method. Clin Chem Acta.

[18] International Committee for Standardization in Hematology (1967). Recommendation for haemoglobimonetry in human blood. Br J Haematol.

[19] Marx G (2003). Evolution of fibrin glue applicators. Transfus Med Rev.

[20] Nagelschmidt M (1999). Endoscopic use of fibrin adhesives: problems when injecting through long catheters. Surg Endosc.

[21] Eder F, Meyer F, Nestler G, Halloul Z, Lippert H (2005). Sealing of the hepatic resection area using fibrin glue reduces significant amount of postoperative drain fluid. World J Gastroenterol.

[22] Shimada J, Mikami K, Nishiyama K, Satoh S, Wada Y, Kimura T (1995). Closure of leaks by fibrin gluing. Effects of various application techniques and temperatures. J Cardiovasc Surg (Torino).

[23] Bonatti H, Bodner G, Obrist P, Bechter O, Wetscher G, Oefner D (2003). Skin implant metastasis after percutaneous radio-frequency therapy of liver metastasis of a colorectal carcinoma. Am Surg.

[24] Llovet JM, Vilana R, Brú C, Bianchi L, Salmeron JM, Boix L (2001). Barcelona Clínic Liver Cancer (BCLC) Group. Increased risk of tumour seeding after percutaneous radiofrequency ablation for single hepatocellular carcinoma. Hepatology.

[25] Jiao LR, Williamson RCN, Habib NA (2003). Radiofrequency comes of age in liver surgery: ablation technique and adjunct to resection. HPB.

